# Prolonged asystole during REM sleep: A case report and review of the literature

**DOI:** 10.1016/j.hroo.2022.07.007

**Published:** 2022-07-22

**Authors:** James R. Sampognaro, Andreas S. Barth, Jonathan C. Jun, Jonathan Chrispin, Ronald D. Berger, Charles J. Love, Courtney Eddy, Hugh Calkins

**Affiliations:** ∗ Division of Cardiology, Department of Medicine, The Johns Hopkins University School of Medicine, Baltimore, Maryland; † Division of Pulmonary and Critical Care, Department of Medicine, The Johns Hopkins University School of Medicine, Baltimore, Maryland

**Keywords:** Asystole, Cardiac monitors, Electrophysiology, Nocturnal sinus pauses, REM sleep

**Key Findings**

▪ A 63-year-old man without obstructive sleep apnea who reported dizziness with running was found to have frequent nocturnal episodes of asystole, predominantly during rapid eye movement (REM) sleep, lasting up to 12.1 seconds. This presentation is consistent with a rarely described syndrome called “REM-related asystole.”
▪ Members of the Heart Rhythm Society were surveyed as to how they would manage this clinical scenario, and many reported having encountered similar cases with various treatment approaches.
▪ We review 22 prior cases of REM-related asystole described in the literature. Most of these patients had no significant cardiovascular past medical history and had normal daytime cardiovascular testing. However, nearly all were implanted with pacemakers.
▪ Little is known about the mechanism of REM-related asystole, its prevalence, or its long-term outcomes. Although the most recent ACC/AHA/HRS guidelines recommend against pacing in patients for the indication of sleep-related sinus pauses, more research is needed to elucidate the prognosis of this condition and inform future guidelines.

## Introduction

In the general population, sleep is associated with bradyarrhythmias owing to increased vagal tone.^1^, ^2^, ^3^, ^4^ Up to 5.7% of asymptomatic, healthy patients have been reported to have nocturnal sinus pauses >2 seconds on cardiac monitoring, with the most prolonged pause of 2.8 seconds in these studies.^1^^,^^2^^,^^5^^,^^6^ Such asymptomatic pauses are typically considered physiologic, not requiring intervention. In contrast, between 10% and 15.6% of patients with known obstructive sleep apnea (OSA) have been reported to have sinus pauses >2 seconds during sleep studies, with the most extended pause of 13 seconds.^7^, ^8^, ^9^ For this reason, the 2018 ACC/AHA/HRS guidelines give a Class I recommendation to screen for symptoms of OSA in patients with nocturnal bradycardia, with subsequent confirmatory testing as directed by clinical suspicion.^10^ If OSA is confirmed, a Class I recommendation is given for treatment directed specifically at OSA, since continuous positive airway pressure has been shown to effectively suppress these bradyarrhythmias during sleep.^9^^,^^11^ These same guidelines, however, give a Class III recommendation against pacing in patients with sleep-related sinus pauses unless other indications for pacing are present. We present a case of a 63-year-old man without OSA who was found to have frequent episodes of asystole, predominantly during rapid eye movement (REM) sleep, lasting up to 12.1 seconds, and we review what is currently known about this condition.

## Case report

A 63-year-old man with a past medical history only significant for treated Lyme disease and no significant family history presented to his primary care provider for several months of brief dizziness episodes that would occur while running, often after episodes of belching. His primary care provider recommended that he wear a Zio XT monitor for 14 days to evaluate for potential cardiac causes of dizziness.

The patient’s monitor report showed a predominant underlying sinus rhythm with an overall average heart rate of 50 beats per minute (bpm). However, 971 sinus pauses >3 seconds were observed, with the majority occurring between 10 PM and 6 AM. Of these, the 2 longest pauses were 12.1 and 8.8 seconds, both occurring between 1 and 2 AM. The monitor also detected 4 runs of supraventricular tachycardia (SVT), with a longest of 4 beats at 138 bpm. In addition, there were 11 patient-initiated triggers during symptomatic events, which were associated mainly with normal sinus rhythm, sinus tachycardia, and sinus slowing with junctional rhythm (76–79 bpm). There were no patient triggers associated with sinus pauses.

Given these findings, he was urged by a covering physician to present to his local emergency room for further evaluation and monitoring. His electrocardiogram (ECG), echocardiogram, and other laboratory work were largely unremarkable, although he was noted to have a 3.5-second sinus pause during sleep while inpatient. Given a lack of symptomatic bradycardia, he was discharged and told to follow up as an outpatient.

He ultimately established care with the cardiac electrophysiology clinic at Johns Hopkins Medicine. Owing to suspicion of OSA as a potential cause of asystole, he was referred for an in-lab polysomnography, which included assessment of sleep staging, oximetry, respiratory airflow and effort, movement, and a single-lead ECG. During this sleep study, the ECG demonstrated 40 episodes of asystole lasting 2–6 seconds, mostly occurring during REM sleep. Notably, there was no significant degree of sleep apnea. He was awoken from sleep during one episode to ensure safety, and he reported no symptoms. After continued events, he was advised to present to the emergency room, which he declined. For daytime cardiac evaluation, an exercise stress test was ordered to evaluate for chronotropic incompetence. He exercised for 11 minutes, and his heart rate appropriately increased from 49 to 139 bpm. However, he did have abrupt sinus slowing and a junctional beat during the initial portion of this test. A subsequently performed tilt table test was negative. He later underwent testing for genetic variants associated with arrhythmias and cardiomyopathy, which identified him only as a carrier for *FKRP*, a gene associated with autosomal recessive muscular dystrophy.

**Figure 1:**
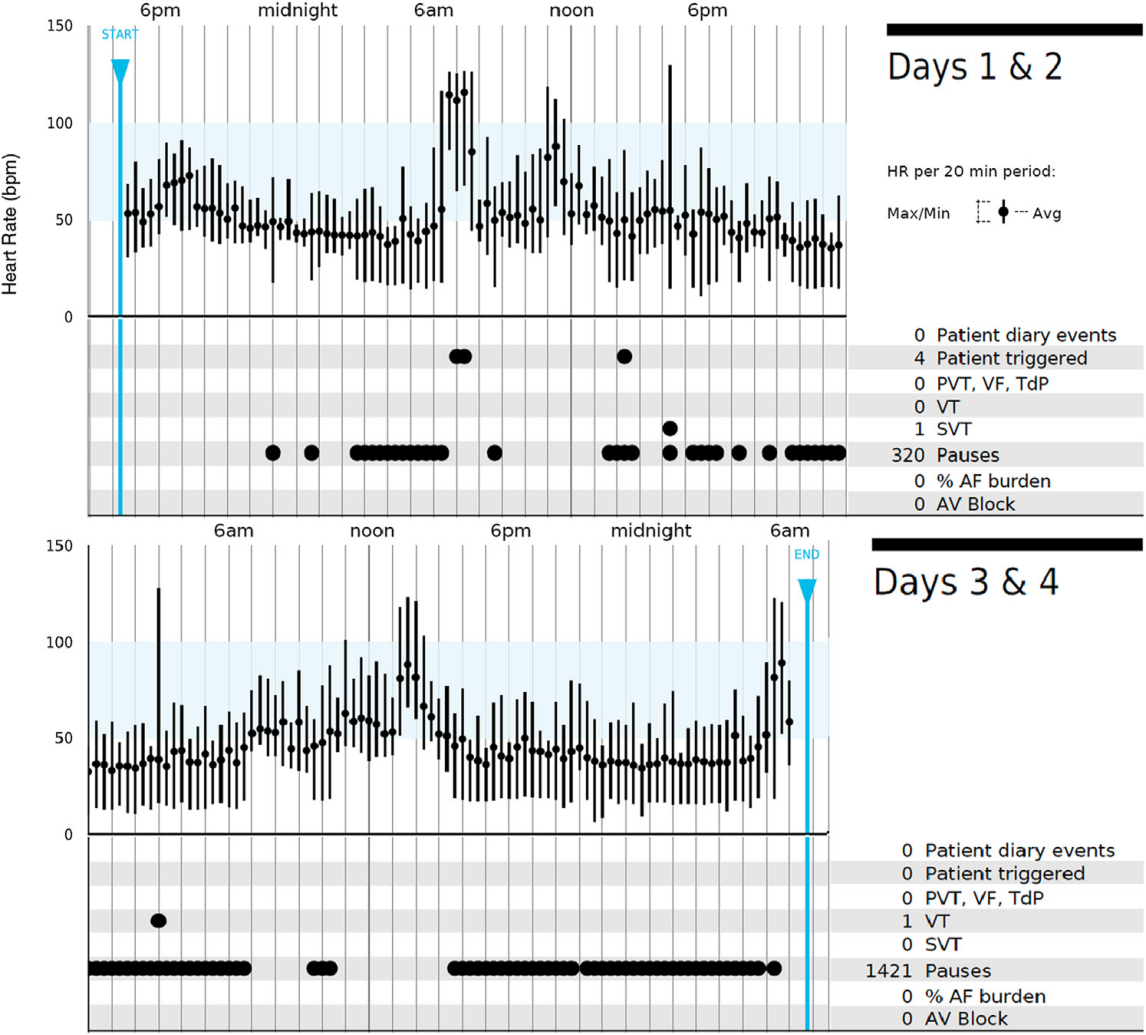
Event log adapted from patient’s Zio XT report.

**Figure 2:**
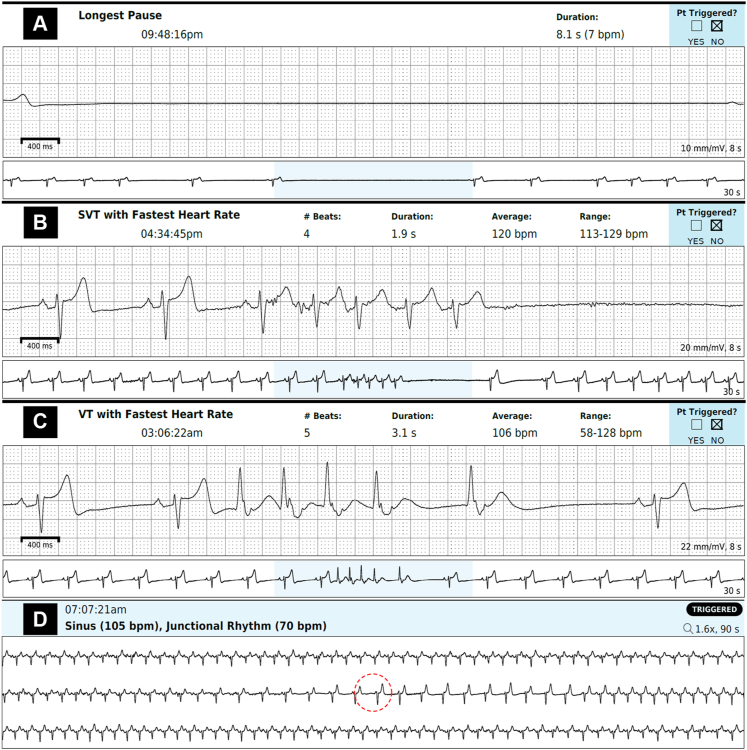
**A–D:** Key tracings adapted from patient’s Zio XT report. The red dashed circle in panel D indicates the timing of a patient trigger during a symptomatic event. SVT = supraventricular tachycardia; VT = ventricular tachycardia.

To be certain that this was not a self-terminating condition, he underwent a repeat 48-hour Zio XT monitoring weeks later, which again showed an underlying sinus rhythm with an average heart rate of 50 bpm. Additionally, 1741 sinus pauses occurred, predominantly at night, with longest pauses of 8.1 and 8.0 seconds occurring around 10 PM (Figure 1, Figure 2A). He also had a 4-beat run of SVT (maximum rate 129 bpm, Figure 2B) and a 5-beat run of nonsustained ventricular tachycardia (maximum rate 128 bpm, Figure 2C). Again, patient triggers were associated with normal sinus rhythm, sinus tachycardia, and sinus slowing with junctional rhythm (70 bpm, Figure 2D); none of the 4 patient triggers were associated with sinus pauses.

We reviewed the literature (discussed in the following section) and found little data on the prognosis of REM-related sinus pauses. Given guidelines against pacemaker implantation for sleep-related sinus pauses, our group initially agreed that it would be best to refrain from permanent pacemaker (PPM) implantation. However, to survey broader expert opinion, we posted a brief description of our patient’s case in the Heart Rhythm Society (HRS) Member Open Forum. We asked whether other HRS members would implant a PPM in this scenario. Within a week of posting, we received responses from 27 different cardiac electrophysiologists from around the world. Of these responses, we categorized 2 responses as “yes,” 8 as “maybe,” and 17 as “no” (Table 1). The most frequently cited rationale behind “no,” consistent with our internal discussions, was that his pauses appeared to be asymptomatic. Interestingly, these respondents mentioned encounters with 15 patients with findings similar to ours. Of those 15 patients, 8 were followed without treatment, 5 received a PPM, and 2 underwent cardioneural ablation.

**Table 1:** Heart Rhythm Society member open forum responses

Resp.	Rec. PPM?	Comments
1	Maybe	Had a similar pt for 10 years without issue. Highlighted need for more data
2	No	Not without symptoms
3	Maybe	Highlighted need for topic to be mentioned in guidelines
4	No	Consider CNA
5	No	No PPM or CNA without symptoms
6	No	Had 2 similar pts who developed pauses and symptoms while awake, chose CNA with good results
7	No	Not without symptoms
8	No	No PPM regardless of pause length
9	Maybe	Had a similar pt with 12-second pauses and shaking, would frequently fall out of bed. PPM resolved symptoms. Highlighted need for more data/sleep EEGs.
10	No	Consider CNA if daytime symptoms
11	No	CNA if severe daytime bradycardia, or PPM if severe nocturnal pauses with pause-dependent wide QRS ventricular escape beats or NSVT
12	No	Had 3 similar pts (2 complete HB, 1 sinus pauses) whose arrhythmia burden improved with SSRI (chosen for pro-adrenergic and pro-serotoninergic actions)
13	Maybe	Had a similar pt in whom PPM resolved diurnal “brain fog.” Recommended broad definition of “symptomatic.” Wondered about thrombotic risk with long pauses
14	No	Commented that vagotonia from running is not responsible since REM vagal tone is less predominant. Suggested removing culprit medications
15	Maybe	Had 2 similar pts without issue. Highlighted need for more guidance in literature
16	No	Recommended de-training in athletic pts but recognized difficulty of adherence
17	No	Not without daytime symptoms, recommended extended monitoring to detect association with daytime symptoms
18	Yes	Had a similar pt whom he implanted after discussing with Dr Guilleminault (author of 2011 review)^13^ owing to theoretical risk of sudden cardiac death
19	No	Had a pt with pause of 13.8 seconds, no issue with 7 years of follow-up
20	No	Not without symptoms
21	No	Would not implant without symptoms but wondered about subclinical brain damage during prolonged asystole
22	No	Not without presyncope or syncope
23	Maybe	Had 2 similar pts found on cardiac monitor after cryptogenic stroke. Wondered if pauses caused stroke and if related cerebral hypoperfusion is damaging in elderly pts. Highlighted need for outcomes research
24	Maybe	Had a similar pt in whom an *HCM4* mutation was discovered and subsequently underwent PPM implantation. Recommended genetic testing.
25	Yes	Had a similar pt who received PPM, AAI mode. Later developed more exertional fatigue on runs; AAIR mode resolved symptoms
26	Maybe	Highlighted difficulties with lack of prognostic data. Mentioned that a colleague implants PPM for pauses >8 seconds
27	No	Not without daytime symptoms

CNA = cardioneural ablation; EEG = electroencephalogram; HB = heart block; NSVT = nonsustained ventricular tachycardia; PPM = permanent pacemaker; Pt(s) – patient(s); Rec. = recommended; REM = rapid eye movement sleep; Resp = respondent; SSRI = selective serotonin release inhibitor.

Shortly after this workup and discussion, however, our patient had a syncopal episode while running and sustained an ankle injury. Unfortunately, he did not have a monitor in place during this incident, so we do not know his heart rhythm at the time of syncope. However, with the development of new exercise-related syncope in the setting of profound sinus pauses (predominantly but not exclusively nocturnal), symptomatic episodes of sinus slowing with junctional rhythm, and runs of SVT and nonsustained ventricular tachycardia, we ultimately decided with the patient to pursue pacemaker implantation.

## Discussion

Our patient’s history is consistent with a condition, first described in 1984, referred to as “REM-related bradyarrhythmia syndrome.”^12^^,^^13^ This term encompasses both REM-related asystole (as seen in our patient) and REM-related complete heart block. Although sinus bradycardia during non-REM sleep can be attributed to a physiologic increase in parasympathetic tone, a REM-specific bradyarrhythmia warrants a different explanation. Unlike non-REM sleep, REM sleep is typically associated with decreased vagal tone and concomitant surges in sympathetic activity during phasic events, resulting in increased heart rates.^13^ Thus, REM-related bradyarrhythmias may result from a dysfunction in autonomic modulation, with a nonphysiologic predominance of vagal tone compared to sympathetic activation during this phase of sleep. It is unclear whether this imbalance primarily results from abnormally heightened vagal tone during REM sleep or from a withdrawal of sympathetic input during phasic events. Additionally, it is unknown whether this abnormal modulation results from altered central influences on the autonomic nervous system or from an abnormal baroreceptor reflex.^13^

REM sleep–related asystole has rarely been reported in the literature, and this condition’s prevalence and natural course remain unknown. We found 22 similar cases of REM-related asystole in the literature, of which 18 were described in a 2011 review^13^ and 4 have been described in subsequent case reports.^14^, ^15^, ^16^, ^17^ The overall characteristics of these patients in addition to our patient are summarized in Table 2, and the individual case characteristics are presented in Table 3. The vast majority of these patients were male (87%), and the average age at diagnosis was 30.5 years. Our case describes the eldest patient (63 years old) known to be diagnosed with this condition. Of these 23 patients, only 1 had any significant cardiovascular past medical history (hypertension), and none had illicit drug use.

**Table 2:** Summary of rapid eye movement sleep–related sinus arrest cases (N = 23 patients)


Age, years	30.5 (± 12.0)
Male	20 (87.0%)
No prior cardiovascular history	22 (95.7%)
Longest pause on cardiac monitor, seconds	10.4 (± 2.6)
Longest pause during REM on PSG, seconds	7.6 (± 3.1)
Pacemaker implantation	19 (82.6%)

For continuous variables, numbers are represented as % (± SD); for categorical variables, numbers are represented as n (%).

PSG = polysomnography; REM = rapid eye movement sleep.

**Table 3:** Individual cases of rapid eye movement sleep–related sinus arrest

Year, ref.	Age, sex	PMH	Symptoms	Normal diurnal cardiac eval.	Abnormal diurnal cardiac eval.	Cardiac monitor, longest pause (s)	PSG, longest pause (s)	Device?
1984^12^	30, F	None	Syncope after awakening, CP, palpitations	ECG, EST, TTE, Cath, EPS, ANS	None	9	5	PPM
1984^12^	27, M	None	CP, dizziness	ECG, EST, Cath, ANS	None	8	5	PPM
1984^12^	35, M	None	Syncope after awakening, CP	ECG, EST, Cath, ANS	None	7	6	PPM
1984^12^	NR, M	None	CP	ECG, EST, EPS, ANS	None	8	6	None
1999^19^	27, M	None	Syncope w/ nocturnal BMI, CP, dizziness	EST, TTE, EPS, Tilt, ANS	None	7.2	5	None
2004^21^	23, M	None	None	ECG, EST, TTE, Tilt, EPS	None	11.8	5.5	PPM
2007^22^	54, M	BMI 37	CP, presyncope w/exercise	EST, TTE, ANS	None	7.0	6.7	PPM
2007^22^	41, M	None	CP	TTE, Cath, Tilt	None	8.2	11	PPM
2011^13^	29, M	None	Presyncope w/ exercise	ECG, Cath, EST	None	12	10	PPM
2011^13^	23, M	None	Presyncope w/ early AM urination	ECG, Cath, EST	None	15	7	PPM
2011^13^	20, F	None	Syncope w/menstruation	ECG, Cath	None	11	8	PPM
2011^13^	22, M	None	Unknown	ECG, Cath, EPS	None	13	7	PPM
2011^13^	24, M	None	Presyncope w/exercise	ECG, Cath, EPS	None	12	10	PPM
2011^13^	21, M	None	Presyncope w/intercourse	ECG, Cath, EPS	None	14	6	PPM
2011^13^	19, M	None	Malaise w/exercise	ECG, Cath, EPS	None	12	5	PPM
2011^13^	18, F	None	Malaise w/exercise	ECG, Cath, EPS	None	12	10	PPM
2011^13^	23, M	None	Presyncope after awakening	ECG, Cath, EPS	None	9	9	PPM
2011^13^	24, M	None	Presyncope w/exercise	ECG, Cath, EPS	None	12	8	PPM
2012^14^	43, M	None	None	None	Brady. on ECG	13.8	5.1	None
2015^15^	36, M	None	Syncope after awakening	ECG, TTE	EPS with pauses up to 4.1 s	7.8	8.6	PPM
2018^16^	49, M	BMI 34, HTN, pAF	Dyspnea, snoring	EST	TTE w/LVH, diastolic dys.	5.5	5.5	PPM
2022^17^	21, M	None	Presyncope after awakening	ECG, TTE, EST, EPS	None	10.9	19.5	PPM
Current	63, M	Lyme (treated)	Syncope, presyncope w/exercise	ECG, TTE, EST	None	12.1	6	PPM

ANS = autonomic nervous system study (eg, Valsalva ratio, heart rate variability); BMI = body mass index; Cath = cardiac catheterization (right- vs left-sided not specified); Brady. = bradycardia; CP = chest pain; dys = dysfunction; ECG = electrocardiogram; EPS = electrophysiology study; EST = exercise stress test; Eval. = evaluation; LVH = left ventricular hypertrophy; F = female; M = male; NR = not reported; pAF = paroxysmal atrial fibrillation; PMH = past medical history; PSG = polysomnography; Ref. = reference; Tilt = tilt table test; TTE = transthoracic echocardiogram.

The patients underwent initial evaluation owing to a range of symptoms, including daytime chest pain, daytime dizziness, and occasional presyncopal or (in 4 cases) syncopal events near the time of awakening. Daytime cardiac evaluations were almost universally normal, including ECGs, transthoracic echocardiograms, diagnostic electrophysiology studies, cardiac catheterizations, and tilt table tests.^16^ In each case, the patient was diagnosed with REM-related asystole via a sleep study obtained in response to nocturnal pauses detected on prolonged cardiac monitoring. The longest pauses in these patients during sleep studies ranged from 5 to 19.5 seconds. REM-related asystole was not associated with sleep apnea in these patients.

To investigate a possible overlap between REM-related asystole and sinus node dysfunction (SND), genetic testing was suggested by a member of the HRS Online Forum who had identified a similar patient with SND harboring a pathogenic variant in the cardiac pacemaker channel HCN4. Although the 2018 ACC/AHA/HRS guidelines state that genetic testing is not routinely indicated for SND,^10^ the newer 2022 European guidelines note that genetic testing may be considered as part of the diagnostic evaluation for patients with familial or isolated but otherwise unexplained SND, or with SND and concomitant atrial fibrillation, cardiac conduction disease, structural heart disease, or extracardiac syndromal features, especially in the setting of a positive family history.^18^ In line with the notion that most sinus node disease is acquired and that there is low sensitivity for detecting a causal genetic variant, no pathogenic mutation was identified in our patient.

Given the relatively scant data on REM-related asystole, there has been no consensus on how to treat these patients. Multiple drugs have been trialed, with variable success. Atropine (used in 2 patients) and protriptyline (1 patient) decreased episodes of REM-related sinus arrest,^12^ but amitriptyline (1 patient) was ineffective.^19^ These drugs were discontinued in each case owing to adverse effects. Of the 22 patients previously described, 18 ultimately underwent PPM implantation. Long-term follow-up was reported in only 7 of these patients, but all remained asymptomatic after implantation.

Although PPM implantation was performed in 18 of these prior cases, 2018 ACC/AHA/HRS pacing guidelines recommend against pacing for the indication of sleep-related sinus pauses. These guidelines recommend a sleep apnea evaluation, but REM-related asystole as a condition is not mentioned. In contrast, the 2021 European Society of Cardiology (ESC) pacing guidelines recommend that these patients be evaluated for both sleep apnea and REM-related bradycardia.^20^ These guidelines state that most patients with REM-related asystole have received PPMs but acknowledge that there is little evidence for this practice. More generally, the 2021 ESC guidelines give a Class IIb recommendation for pacing in patients who have experienced syncope and asymptomatic sinus pauses >6 seconds, which would apply to our patient. Notably, however, only 5 of the patients in the REM-related asystole literature presented with syncope. Those described in prior cases and in the HRS online forum generally displayed nonspecific symptoms, and REM-related asystole was discovered incidentally. Interestingly, a common anecdote from the literature and HRS forum was that vague presenting symptoms often improved after PPM implantation. One athlete (not included in the literature review owing to a lack of sleep study) with incidentally discovered nocturnal pauses up to 15 seconds even reported increased energy and significantly improved running times after PPM implantation, despite being reportedly asymptomatic before implantation.^4^

As a result of underdiagnosis and underreporting, we do not have data on important prognostic questions regarding this condition, such as whether it may lead to increased stroke risk or unexplained cases of sudden cardiac death during sleep. As a result, it is clear from our literature review and HRS online forum survey that the management decisions regarding these patients have been quite variable. As wearable cardiac monitors become increasingly widespread, we will encounter this finding more frequently, and we will need to ensure that we are treating patients appropriately. Thus, more research is necessary to describe the prognosis of this condition, determine the best management, and inform evidence-based guidelines.
